# A systematic review of the neurobiological aspects of memory in the aging process

**DOI:** 10.1590/S1980-57642011DN05040009

**Published:** 2011

**Authors:** Eduardo Moreira de Oliveira, Priscilla Tiemi Kissaki, Tiago Nascimento Ordonez, Thaís Bento Lima-Silva

**Affiliations:** 1 Bacharel em Gerontologia - Escola de Artes, Ciências e Humanidades da Universidade de São Paulo, São Paulo SP, Brazil. Pesquisadores do Núcleo de Estudos no Envelhecimento Cognitivo e Núcleo de Estudos em Gerontologia, EACH-USP, São Paulo SP, Brazil.; 2 Pós-graduada em Neurociências pela Faculdade de Medicina do ABC - Mestranda em Neurologia, pelo Departamento de Neurologia Cognitiva e do Comportamento - Faculdade de Medicina da Universidade de São Paulo, São Paulo SP, Brazil.

**Keywords:** memory, neurobiology, neuropharmacology, aging

## Abstract

A systematic review of the neuroanatomical literature was performed to determine the neuropharmacological aspects most relevant to the study of memory processes. Articles were retrieved using the search terms "biology of memory", "memory and aging", "memory impairment", "elderly and memory," and their equivalents in Portuguese. Of the studies surveyed, five studies dealt with epidemiological and demographic issues, 12 were clinical trials i.e. were based on testing and implementation of instruments in human subjects, 33 studies were basic research involving studies of mice, rats and non-human primates, and biochemical and *in vitro* trials and finally, 52 studies were literature reviews or book chapters which in our view, fell into this category.

**Conclusions:**

The work sought to highlight which neural networks are most involved in processing information, as well as their location within brain regions and the way in which neurotransmitters interact with each other for the formation of these memories. Moreover, it was shown how memory changes during the normal human aging process, both positively and negatively, by analyzing the morphological alterations that occur in the brain of aging individuals.

## Abstract (pt)

Buscou-se verificar na literatura os aspectos neuroanatômicos e neurofarmacológicos mais relevantes no estudo dos processos de memória, através de revisão sistemática. O levantamento bibliográfico foi realizado utilizando-se os termos "*biology of memory", "memory and aging", "memory impairment", "elderly and memory*", e seus correspondentes em português. Dos estudos levantados, cinco estudos tratavam sobre questões epidemiológicas e demográficas; 12 estudos eram de base clínica, ou seja, as pesquisas ocorreram com base em testes e aplicação de instrumentos em seres humanos; 33 estudos foram oriundos da pesquisa básica, envolvendo pesquisas com camundongos, ratos e primatas não humanos, e ensaios bioquímicos e *in vitro*; e, por fim, 52 estudos são revisões da literatura ou capítulos de livros que, a nosso ver, enquadram-se nesta categoria.

**Conclusões:**

Buscou-se dar destaque para quais redes neurais estão mais envolvidas no processamento das informações, bem como sua localização dentro das regiões cerebrais e a forma com a qual os neurotransmissores interagem entre si e atuam para a formação destas memórias. Ademais, mostrou-se como a memória se altera ao longo do processo de envelhecimento humano normal, negativa e positivamente, analisando as alterações morfológicas que ocorrem no cérebro do indivíduo que envelhece.

## Introduction

The aging of the world's population stems from a falling birth rate allied with rising life expectancy.^1,2^ This phenomenon has resulted in a shift in the age structure of populations, with a disproportionate increase in the number of elderly individuals (60 years of age or older in developing countries, according to World Health Organization definition) compared to other age groups.^3,4^ Among the physiological changes associated with the aging process is a decline in some cognitive abilities with memory numbering among them.^5-8^ According to Izquierdo,^9^ memory can be defined as the acquisition, storage, and retrieval of information which is presented to us throughout the life course. Thus, our collective memory ultimately shapes our personality and has social implications since cultural characteristics such as beliefs and customs mark the identity of peoples with memories which are shared by members of the group.^9,10^

In addition, memory is linked to our social integration such as through interaction among people and knowledge of rules and laws, as well as of facts and events. Memory also plays a key role from an evolutionary standpoint because it is essential for reproduction and survival, for example in remembering food foraging sites or the imminent decision for fight or flight.^11,12^

The events outlined above are generally emotionally loaded, both positively and negatively, and are the product of neurochemical changes and interactions with earlier memories, consequently increasing the likelihood of storage of this information.^12,13^

"Memories" in the plural sense as defined by Lent,^14^ can be classified in terms of retention time and their nature, with respective types and subtypes. Regarding retention time, memories can be defined as: *very short term* (lasting only a few seconds)*; short term* (up to three hours); *and long term* (lasting days or years). Concerning the nature of memory, this can be classified as: *explicit* (or declarative); *episodic* (has temporal reference); *semantic* (involves temporally unrelated concepts); *implicit* (or non-declarative); *perceptual representation* (represents images with unknown meaning); p*rocedural* (habits and rules); *associative* (classic and operative conditioning); *non-associative* (habituation or sensitisation of the stimulus); and *operational* (or working memory).

Although retrieval of memories requires, obligatorily, that the information has been acquired at some point, its mere acquisition does not make this information a memory per se. The information only becomes a memory when information is learned, stored^11^ and subsequently retrieved.^9^ Thus, learning by information acquisition constitutes the first step toward the formation of memory.

Information acquisition can occur in a variety of ways, albeit through empirical experiences, stimuli captured by our sensory and somesthetic systems, or even through situations created in our own thoughts.^11^

Indeed, any stimuli that pass through the memory-related neural systems may be selected for subsequent consolidation. The process of information selection is important to enable systems involved in memory to refine, amongst all the complexity of information it receives, which items are most relevant based on the focus given to the information, emotions involved with it, among other as yet unknown systems of refinement.^11,15^

Defining specific brain regions or circuits dedicated to memory is hard given these processes are distributed throughout the brain, but some circuits have more affinity for certain stages of the memory formation process.^11,13^

The hippocampus, a structure located bilaterally within the medial temporal lobe, has the function of consolidating information it receives, transferring this to the appropriate cortical regions or for temporary storage of copies of cortical enagrams via interaction with other regions of the hippocampal formation, particularly the entorhinal cortex, through its connections with Ammon's horns and the dentate gyrus of the hippocampus. According to Cassini,^16^ there is evidence that with time, hippocampal activity decreases in response to the retrieval of distant memories. On the other hand, an increase in cortical activity takes place. This suggests that memory progressively, dynamically and in a plastic manner, becomes independent of the hippocampus and more dependent on cortical regions, a process called Systemic Consolidation or Reorganization of Memory.^16,17^

The dentate gyrus region of the hippocampus, as well as CA1 and CA3 regions, perform an important role in contextualizing aversive memories.^18^ The dentate gyrus appears to be involved in the separation between similar spatial memories. The CA3 region seems to be important in the processes of storage and retrieval of associative memories, while the CA1 regions appears to be related to recognizing new items in context and to information retrieval processes, contributing to the temporal aspects of memory.^18-20^

In addition, regions such as the medial and inferior temporal gyrus play a key role in memory, housing the language lexicons which allow use to describe everyday objects and situations. The posterior region of the parietal cortex stores information on the place information, working in conjunction with the medial pre-frontal cortex which orients us on distance between locations.^11^

The involvement of subcortical nuclei such as the diencephalic nuclei, septal nuclei of the prosencephalic base and mammillary bodies of the hypothalamus, in memory processes remains unclear, although damage in these latter bodies can result in episodic memory impairment in rats.^21^ However, the intimate relationship that these nuclei have with the hippocampus, whether through the connections via fibers of the fornix or cholinergic projections, indicates participation of these structures in memory consolidation, or as a co-adjuvant in this process.^11^

While the predominant role of the brain structures outlined is the storage of information, other structures are pivotal for this process to take place, namely, the structures making up the so-called to limbic system.^22^

The limbic system, originally referred to as "Broca's lobe" or "Papez's Circuit",^22^ consists of a series of structures located mainly in the temporal lobes, and includes structures such as the hypothalamus, amygdala, basal ganglia, pre-frontal area of the cortex, cerebellum, septum and nucleus accumbens. Nevertheless, this is only one definition of "limbic system", since there is no consensus in the literature regarding the regions involved in the emotional circuitry modulating memory.^23,24^

These structures are intimately related with emotions to be aggregated to information received in so-called "reward pathways" and "fear circuit".^25^ The importance of emotions in memory processes arises since events intertwined to strong emotional reactions have a greater likelihood of being stored with a single exposure to events.^13,26-29^

**Figure 1:**
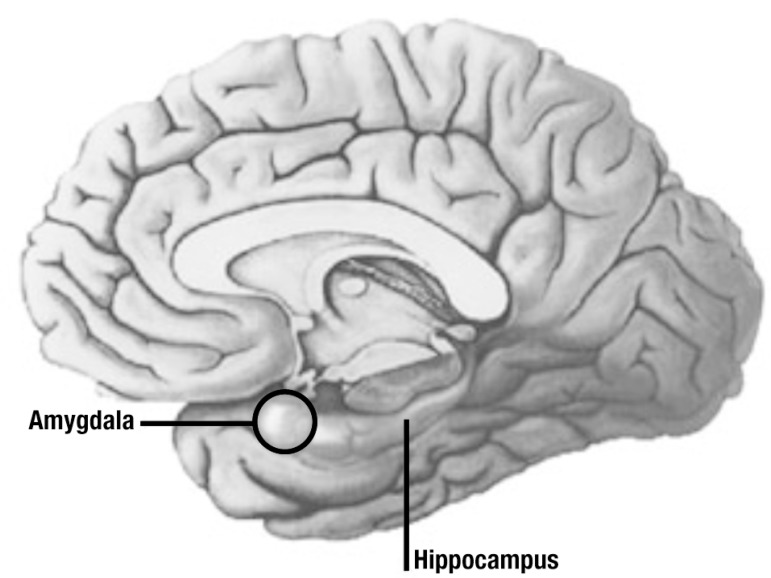
Intimate relationship between amygdala and hippocampus.

A number of studies have shown that the amygdala (Figure 1), also located in the temporal lobe, has an important role in fear conditioning.^30,31^ According to the authors, information derived from the cerebral cortex and hypothalamus enters the amygdaloid complex via the basolateral nucleus and is transmitted to the central nucleus of the amygdala and subsequently distributed to regions of the brain trunk and hypothalamus which orchestrate defense behavior.^32,33^

Since activation of the fear pathway plays a relevant role for the survival of the organism, the formation and storage of this information can lead to long term potentiation (LTP) in the amygdala.^34,35^ LTP can be defined as the increase in post-synaptic responses over a long period, after brief repetitive stimulation of pre-synaptic afferents, in which the resulting synaptic modifications are retained for a long period after the transcription of genes and synthesis of proteins, resulting in greater efficacy of synaptic transmission.^36-38^

While some authors hold that the amygdala has a role in forming the association between stimulus and response to fear conditioning,^39-41^ other studies propose a modulating role played by the amygdala on the acquisition and storage of information in other structures depending on the integrity of the connections between it and the hypocampus.^42-44^

The basolateral amygdala nucleus can influence hippocampal activity by means of direct projections to this structure, or via the entorhinal cortex, with involvement not only in the processes mediating long-term consolidation, but also in the acquisition and codifying of information.^44^ The processes of memory consolidation can be regulated by circulating adrenalin, noradrenalin, ACTH, among others, acting on the basolateral complex of the amydala.^45^

Quevedo et al.^45^ reported that in animal studies, the amygdala was found to regulate the processes of synaptic plasticity important for the formation of memory in other regions of the brain such as the hippocampus.

Despite the amygdala and hippocampus complex being independent memory systems, they interact when the information has aggregated emotion.^46^ Another region of the limbic system which forms the neural substrate for emotional experience is the Nucleus Accumbens.^10^ This region comprises the prosencephalic base together with ventral portions of the caudate and putamen, constituting the ventral striatum.^47,48^

Despite the fact that the influence of the nucleus accumbens on the formation of memory has been little explored, some studies have implicated participation of this area both in spatial memory and associative memory by aversive stimulation.^49,50^ Moreover, this nucleus is known to be involved in the so-called reward pathway, being associated to pleasurable stimulus. This structure of the prosencephalic base receives dopaminergic afferents from the VTA and glutamatergic afferents from regions including the pre-frontal cortex, amygdala and hippocampus.^51^

The nucleus accumbens has a central region in its structure called the nucleus and another denominated peripheral. The nuclear region receives noradrenergic afferents originating from the Nucleus Tractus Solitarius (NTS) and also afferents from the hippocampus and amygdala, which process the newly acquired information. The projections from the basolateral nucleus of the amygdala provide affective components of experiences, and the projections from the region of the ventral subiculum of the hippocampus supply information on the contextual characteristics of the environment. The noradrenalin released by the afferents of the Nucleus Tractus Solitarius in the shell region of the nucleus accumbens exerts an important modulatory action on memory formation processes, regulating synaptic activity and facilitating mnemonic processes.^52^ Further, the increase in noradrenalin levels can lead to enhanced memory performance, whereas the blocking of noradrenergic receptors has the opposite effect, indicating an important role of noradrenalin in the processes of emotional memory.^53^

Both the amygdala and the nucleus accumbens are involved in the emotional processing which the information undergoes. The pre-frontal cortex belongs to this group, representing a region involved in the reward pathway which also receives afferents from the nucleus accumbens. According to Haber & Knutson,^54^ humans display greater activity in certain areas of the pre-frontal cortex depending on the type of reward. Sensory rewards activate more posterior regions of the orbitofrontal cortex whilst abstract rewards tend to activate more anterior regions. Also, rewards tend to activate medial regions of this cortex, proximal to the gyrus rectus, whereas aversive stimuli tend to activate more lateral regions of the orbitofrontal cortex. A study carried out by Machizawa et al.^55^ showed a strong relationship between the left inferior frontal gyrus and the formation of long-term memory, as well as the ventrolateral pre-frontal cortex and the formation of these memories in humans.

Besides the above-mentioned structures, the neurotransmission systems have direct or indirect action on memory processes. As cited earlier, noradrenalin has an important role in the formation of emotional memory in structures of the limbic system, interacting with cortisol during this process.^53^

Another neurotransmitter heavily involved in memory processes is acetylcholine.^56^ The majority of cholinergic efferents stem from the prosencephalic base by neurons of the medial septal nucleus, diagonal band, *substantia innominata* and basal nucleus of Meynert.^57^ These neurons project to the hippocampus, neocortex, parts of the limbic system and amygdala to act in the modulation of processes involved in cognition, and their effects are mediated by metabotropic muscarinic and nicotinic ionotropic receptors. A number of studies have shown the distribution of this receptor type in the hippocampus. its relationship with the formation of memories^58-60^ and induction of LTP.^61^ Acetylcholine also modulates glutamate release in dopaminergic neurons in the VTA, which modulates the reward pathway.^62^

LTP in the hippocampal is important for the formation of long-term memory and involves the activation of AMPA and NMDA receptors in synapses of pyramid cells in the CA1 region of the hippocampus^38^ and in the Schaffer collateral and mossy fiber pathways.^63^

During the initial process of LTP, phosphorylation of the AMPA receptors increases its responsiveness for glutamate but during the maintenance phase of this process, new AMPA receptors are taken to the post-synaptic membrane and, later, due to the activation of other mediators and signalling pathways, structural alterations and increased synaptic contact occur.^38,56^ This process promotes changes in synaptic transmission with long-term duration.

The formation of memories is a slow and complex process for which any failure in any of the mechanism results in non-continuity of the process and consequent loss of information. According to Rossato et al.,^64^ the duration of long-term memories differs among the person's own memories and the mechanisms underlying this are not yet understood. The cited author however, suggested that the effect of long-term aversive memory of rats disappeared after injection of a dopaminergic receptor antagonist (D1) into the dorsal hippocampus, some hours after the aversive stimuli. However, intra-hippocampal injection of a D1 antagonist under the same conditions as the previous experiment resulted in the maintenance of the aversive memory. These effects can be modulated by the brain-derived neurotrophic factor and regulated by the ventral tegmental area (VTA), suggesting that the duration of these memories depends on the dopaminergic connections between the VTA and hippocampus.

Dopamine is most abundant in the striated body, extrapyramidal motor system, some regions of the limbic system and hypothalamus.^56^ In the mesolimbic and mesocortical pathways, cellular bodies are found in the mesencephalon and its fibers project into regions of the limbic system, especially to the nucleus accumbens,^65^ amygdala and to pre-frontal cortex.^56^

According to Vijayraghavan,^66^ dopamine plays a fundamental role in the modulation and performance of working memory, given the vast quantity of dopaminergic receptors found in the pre-frontal cortex. Behavioral studies have shown that blocking these receptors has a detrimental effect on working memory. Corroborating this notion, Cools et al.^67^ suggested that subjects with lower performance on a working memory task had less dopamine synthesis in the striatum, whereas individuals with high performance had greater synthesis.

The consolidation of memory can occur in other brain structures besides the hippocampus. Bermudez-Rattoni^69^ found clinical evidence that patients with lesions in the hippocampus, or ablation of the structure, manifested serious problems consolidating declarative memory but not non-declarative memory. The nucleus accumbens, together with other regions of the cortex, is involved in the consolidation of episodic and recognition memories, while the ventral striatum is important for consolidating declarative memory.

All these memory processes described above remain relatively stable during certain periods of our lives. However, during the course of the aging process, some cognitive changes can occur on a physiological level.

Cognition in humans can be understood as abilities such as memory attention, learning, calculus, language, besides the perceptual modalities plus functional and motor executives.^70,71^ Therefore, memory is one of the abilities which declines with ageing. Nevertheless, the different types of memory can decline in different ways. According to Yassuda,^5^ working, episodic and explicit memories undergo greatest decline. Although the underlying reasons for these changes remain unclear, Bertolucci^72^ suggested an influence of perceptual deficits, difficulties concentrating and attention, besides sluggishness in processing information.

In addition, Mattay et al.^73^ indicated that the pre-frontal cortex is subject to the greatest volume changes during the process of normal aging, with a mean reduction of 5% per decade from 20 years of age, whereas the hippocampal decline is more marked at around 60 years of age. Cognitive aging can be influenced by genetic and biological aspects, as well as factors of a social and cultural nature, thus constituting a multifactorial effect.^74^ Brain weight also changes with a gradual reduction of around 10% between 20 and 90 years of age.^75^

Some abilities of executive functions and also episodic memory undergo linear decline from the age of 20 years. However, accumulated experience can result in an improvement in semantic memory up to the age of 60.76 Therefore, the process of normal aging can result in both declines in some memory types yet enhanced performance in others.

Against this background, both national and international studies will now be described documenting the neurobiological process of memory in normal aging.

The literature search was performed using the terms "biology of memory", "memory and aging", "memory impairment", "elderly and memory", and their equivalent terms in Portuguese. The abstracts from this search were analysed to check whether they met inclusion and exclusion criteria.

The inclusion criteria adopted were articles in Portuguese or English published between 2000 and 2011 held on the databases PsycInfo, PubMed, LILACS, SciELO or ScienceDirect as well as book chapters, dissertations and theses that dealt with issues concerning memory and aging.

Exclusion criteria adopted were studies without information describing the analyses performed, and work financed by private enterprises (to avoid conflict of interest).

In the literature search, a total of 128 studies were initially retrieved (including articles, theses, dissertations and book chapters) related to the main theme "memory and aging". After applying the above-described inclusion and exclusion criteria, 102 studies were selected for inclusion in this review. Of the studies surveyed, five dealt with epidemiological and demographic issues, 12 were clinical trials i.e. were based on testing and the application of instruments in human subjects, 33 studies were basic research involving studies of mice, rats and non-human primates, and biochemical and *in vitro* trials and finally, 52 studies were literature reviews or book chapters which in our view, fell into this category.

### Studies investigating neurobiological alterations in aging and their consequences for memory

The biological bases underpinning memory formation involve the recruitment of proteins that are common in many living organisms, human or otherwise. A significant difference among individuals can be ascribed to the memories of experiences acquired. In this process, the same proteins are activated and mobilized, albeit in different ways, to create traits and memory circuits unique to the individual. According to the perspective of Ramon and Cajal described in 1883,9 memories are structural alterations in synapses that are distinct for each memory, or type of memory, and can be modulated by emotions, level of awareness and states of excitation.

As occurs in all biological systems, from the conception of the individual through until old age, the central nervous system develops in such a way that, up to a given point, the organism increases neuroplasticity at a rapid rate (referred to as maturation). After reaching a given point in development (a juncture not clearly determined and that differs among individuals), this declines along with the ability to learn new information. In addition, the ability to retrieve consolidated memories also begins to diminish.

There are however, some types of memory that apparently do not decline during normal aging. Healthy elderly for instance, do not forget how to write, read or make a cup of coffee. These abilities are associated to implicit or procedural memory and are related to regions of the brain that integrate sensory perceptions, emotional information and motor coordination. These abilities recruit areas such as the neocortex, neostriatum, cerebellum and amygdala.^77^ Amid the complexity of structures and neuronal systems recruited together and unconsciously, the retrieval of implicit memory apparently enhances its consolidation, rendering this type of memory longer lasting.

Explicit or declarative memory is a type of associative, conscious and flexible memory. More labile than implicit memory, it relies on the hippocampal system, the efficacy of neuronal transmission and sometimes on conscious reinforcement of learning. While episodic memory is more vulnerable to the typical neurobiological changes seen in aging, semantic memory seems to be better preserved. For example, in the context of semantic memory, studies show that vocabulary increases throughout the life course, provided the individual is not afflicted by hypertension or other chronic diseases impairing cognition.^78^

Some theories attempt to explain the maintenance of some memories as well as the loss of others. These theories are based on biological concepts such as the formation and maintenance of long-term potentiation and long-term depression (LTD). LTD tends to occur among parallel fibers and Purkinje cells in the cerebellum. Apparently, this pairing reduces the stimulus of inhibitory neurons in deep cerebellar nuclei, increasing the stimulation of excitatory neurons rooted in the cerebellum that then lead to the execution of a movement.^79^ This theory could be related to the sustaining of implicit memory.

LTP on the other hand, takes place in many brain areas and is the only model of electrophysiological change induced by stimuli which can last for days to weeks after a brief afferent stimulation. Since they were first proposed to the present day, this process is the best and most widely accepted description to demonstrate long-term memory.

LTP is a specific kind of neuroplasticity which normally persists for long periods. The formation of LTP in areas such as the hippocampus and amygdala is important for the constitution and maintenance of memories. However, humans suffering significant damage to the medial temporal lobe (including the two areas cited above) present serious changes in the memorization process. A famous case illustrating this effect was the patient H.M, who was submitted to bilateral removal of the temporal lobes in an attempt to treat persistent epilepsy.^80^ Other brain regions involved in memory formation where the LTP process was demonstrated included cortex and septohippocampal projections.

The formation of LTP and maintenance of memory depends more on the integrity of the neurons of the cerebral temporal lobes than their quantity. A common notion holds that significant neuronal loss is associated to aging, and can contribute to memory loss in elderly. This notion arises from the observation that neuron demise takes place as a result of diseases such as encephalic vascular strokes and other types of neurodegenerative diseases. Nevertheless, rigorous anatomic studies have shown no significant loss in neuronal cells occur in the regions of Ammon's horns (particularly CA1 and CA3) or in the dentate gyrus in the hippocampi of humans, monkeys, rats or mice during aging. Moreover, recent studies have shown neurogenesis in structures related to the limbic system such as the hippocampus, amygdala, cortex and striatum in the adult phase, a process confirmed in a number of species of mammal.^80-83^ Therefore, the loss in neuroplasticity seems to be related not to neuronal cell loss but to decline in the efficacy of LTP. This reduced efficacy of LTP can occur in any of the three memory formation processes, namely, it can be related to difficulties in the learning, consolidation and retrieval of information.

### Consolidation of information acquired in long-term memory (LTM)

The consolidation of immediate memory into long-term memory actively engages the hippocampus and entorhinal cortex and this, as explained earlier, is related to the formation of LTP. Some authors emphasize that the medial temporal lobes do not comprise the final repository of memories but rather, are responsible for coordinating the strengthening of neural connections. In fact, storage is related to specific processing sites during the perception of novel information and is therefore interlinked with sensory areas.^27^

The formation and maintenance of LTP requires cellular and molecular events which result in the induction, expression and consolidation of neuronal plasticity. Induction is initiated with cellular depolarization which can be reproduced experimentally using tetanic type electrical stimulation (fired electrical pulses at 100Hz, at 200 ms intervals). The resulting depolarizations are transmitted along the length of the whole axon to reach the axon terminal. At this juncture, neurotransmitters are released and the chemical synapse is established. These stimulations connect proteins of the cytoskeleton such as the integrins-actin system and changes in this system modify the density of post-synaptic dendritic spines.^84^

The stimulations occurring in these axon terminals promote stable changes in the cytoskeleton, which constitute consolidation of LTP, visible microscopically as cross-linking of actin filaments. After these events, an increase in receptor density takes place (particularly glutamatergic) or the mobilization of extra-synaptic areas to the dendritic spicules. This leads to stability of post-synaptic excitatory currents.

The types of receptors recruited for the formation of LTP depend on the brain area in which LTP is being formed. Thus, in the CA1 area of the hippocampus, NMDA type and metabotropic glutamatergic receptors are the target to commence LTP. In the CA3 area, as well as in hippocampus and other areas, an NMDA independent mechanism involving kainate receptors is used. In the amygdala, the late phase of maintenance of LTP appears to depend on serotonin 5HT_4_ receptors. For a comprehensive review on this aspect of LTP, see Izquierdo et al.^38^

The actin network is constantly modulated by endogenous factors that positively or negatively affect the production and maintenance of stable LTP. Positive modulators include brain-derived neurotrophic factor (BDNF) and nerve growth factor (NGF), besides some neurotransmitters such as serotonin, acetylcholine, cannabinoids and hormones e.g. corticosteroids and sexual hormones. The neurotransmitter adenosine however, was described as a potent negative modulator of LTP.^85,86^

In elderly individuals, the functionality of LPT can be affected by interference in induction, expression or consolidation of the process, and also as a result of a decline in positive modulators, or increase in negative modulators. However, no underlying mechanism has yet been defined for these neuropharmacological alterations. For example, the reduction in renal depuration of extracellular adenosine was observed in elderly rats, which may contribute to a decline in LTP. Nonetheless, there is no consensus in the literature concerning the maintenance of the quantity of BDNF and NGF neurotrophins in elderly brain. Possible changes in the neurotransmitter systems and neuromodulators can influence the maintenance of LTP for new information acquired at more advanced ages.

### Studies documenting the learning of novel information - mechanisms for short-term memory (STM)

Three basic characteristics have been postulated in a bid to better define the basic mechanisms of short-term storage: it is transient; 2) it does not require anatomic changes in order to be maintained; 3) it does not require new protein synthesis.

Pharmacological trials using enzyme and genic transcription inhibitors revealed that STM does not involve activation of transcription factors, gene expression or protein synthesis, but does require activation of existing enzyme cascades. Therefore, evocation of this type of memory does not require complex changes in neural substrates of the pathways involved. These memories play an important role in keeping information present, when its definitive storage has yet to be provided for (long-term memory).

Briefly, the stages necessary for the formation of STM in the hippocampus, entorhinal cortex and posterior parietal cortex involve, besides mobilizing glutamatergic receptors, the positive modulation of cholinergic receptors (muscarinic and nicotinic), dopaminergic (D-1) and noradrenergic (β) and negative modulation of GABAergic receptors.

In the hippocampus and entorhinal cortex, for the first 60 minutes after learning, the activation of calcium-dependent protein kinases (CDPKs) is necessary, and in the ensuing 30 minutes, the hippocampus requires the participation of cAMP-dependent protein kinases (second intracellular messenger). Despite CDPK involvement, no phosphorylation of cAMP responsive element binding protein 1 (CREB-1), a genic transcription factor, occurs i.e. no protein transcription takes place.^9,87^

Given this mechanism relies on pre-existing intra-cellular pathways, STM is often spared in elderly. However, consolidation of new information in STM, a process which occurs between 6 and 8 hours after initial learning, is a process involving more stages, leading to LTP formation. In some elderly individuals this process can be more compromised.

### Studies documenting reinforced efficacy of LTP during aging: the role of attention and emotions

An important component reinforcing consolidation of information is attention. The amount of attention applied during the learning process leads not only to improved codification of information but also positively influences its subsequent retrieval. Neuroanatomical and electrophysiological studies show that the brain areas critically relating attention with memory consolidation are the pre-frontal and entorhinal cortex, which send out efferents to the hippocampus. Thus, the greater the stimulation and maintenance of attention, the greater the chances of a given item of information remaining permanently consolidated.

For this description, it is assumed that attention depends on mental and emotional states. Some brain areas have been implicated in the modulation of pleasurable or aversive emotions, which contribute greatly to the consolidation of information created during situations loaded with emotions. The amygdala for instance, is a structure which encompasses more than ten nuclei forming the so-called amygdaloid complex.

Each nucleus receives projections from multiple brain regions such as cortex, thalamus, hypothalamus and encephalic trunk. The cortical and thalamic afferents provide information from sensory areas and memory-related structures (pre-frontal, entorhinal and medial septal cortex). Since the amygdala has access to all this newfound information and has projections extending to structures of the limbic system, this creates the conditions conducive to making associations between current sensory afferents and past experiences.^88^

The basolateral complex of the amygdala is responsible for initiating associative learning. Afferents which carry information regarding conditioned and non-conditioned stimuli form the neocortex, thalamus and hippocampus converge in this complex. Lesions to this structure hamper learning and the expression of fear conditioning.

Fear conditioning occurs when a stimulus becomes associated with danger. In fear conditioning experiments, hitherto neutral conditioning stimuli (CS) become associated with unconditioned aversive stimuli (US). CS can be a sound and/or light whereas US a slight electrical stimulus to the paws. With time, CS alone is able to create behavioral responses of defense or fear, such as freezing (the animal remains static so the predator does not note their presence for instance). Thus, this conditioning allows the animal to detect and avoid situations that represent danger, an essential mechanism for survival. This behavior is acquired quickly and is retained in memory for long periods.^30^

In rats , the circuits mediating fear conditioning consist of afferents related to CS and US, which converge to the basolateral amygdala complex where the information is processed. The information is then carried to the central nucleus from which efferents protrude to the hypothalamus, resulting in the expression of the fear response (such as freezing), autonomous response (such as rise in blood pressure and tachycardia), and endocrine response (such as increased hormone production e.g. of adrenaline and corticosterone - equivalent to human cortisol).

Recent studies have shown that LTP occurs within afferents transmitting CS to the amygdala, and that drugs interfering with this type of plasticity when introduced into the lateral amygdala, also affect fear conditioning. It has therefore been suggested that LTP in this nucleus may represent a mechanism by which memories of CS-UC associations are stored. This hypothesis is also supported by the fact that, in the event of interruption in the synthesis of proteins in the basolateral complex of the amygdala, no consolidation of fear conditioning memory takes place. This finding is consistent with the idea, now widely accepted, that the synthesis of proteins is essential for the conversion of short-term to long-term memory in neurons storing associative memories.^33,50^

In addition to memory enhancement through aversive stimuli, information consolidation can also be enhanced through pleasurable stimuli. The nucleus accumbens plays a key role in this process. There is some evidence of the influence of the nucleus accumbens in the formation of memories in the literature. In 1999, Goldenberg et al.^89^ described a case of declarative amnesia (i.e. spatial, temporal) after hemorrhage in the anterior portion of the basal ganglia region, more specifically the nucleus accumbens region. Other studies have reported involvement in this area in spatial memory as well as associative memory, created by aversive stimuli.^49,50^ In fact, this nucleus receives afferents from the pre-frontal cortex, hippocampus, amygdala, and thalamic nuclei. Together with the known involvement of the nucleus in the reward pathway, it can be inferred that it has a role in the formation of memories, perhaps reinforced by pleasurable stimuli.

The influence of emotions on the modulation of positive and negative factors toward maintaining the efficacy of LTP, represent a broad field for research in neurosciences. The structural plasticity needed for the consolidation process and consequently, for memory retrieval (i.e. the formation of the cited LTP mechanism) is dependent on the development of the central nervous system, a process modulated throughout the life course by nutritional status, oxidative balance, physical and cognitive activities, life style as well as mental states. The degree of LTP impairment during aging can also be influenced by the quantity and quality of stimuli which the brain is subjected to during the aging process. Indeed, individuals submitted to intellectual activities throughout the life course have a lower probability of developing dementia.^90,91^ This correlation was confirmed experimentally in young and old animals submitted to enriched environments (cages with objects, ramps, activity wheels, which offer sensory and cognitive stimuli) that presented behavioral as well as morphological and functional changes in the central nervous system.^92-95^ Hence, improved quality of life with increased social interaction,^96-98^ physical activity,^99^ cognitive stimuli,^100^ sensory stimuli (visual, auditory, tactile, etc.) and the adoption of a healthy balanced diet can, even at older ages, lead to improvements in cognitive functions and memory. Furthermore, these factors may also stimulate genic transcription or reduce their deactivation, which in turn can contribute to the maintenance of LTP^101,102^ and declarative memories.

The present article sought to expound on the main biological aspects involved in the processes of memory and aging. However, it should be noted this review was unable to exhaustively cover all knowledge on the subject, since this theme involves a large array of different approaches. Pathological aspects compromising memory were also not addressed since this was beyond the scope of the study. It should also be noted that some of the studies cited in this article used animals and therefore generalization of findings to humans should be done with caution. The aim of the study was to provide neurobiological descriptions documented to date on the formation of memories. This field of science is still in its infancy, and the present study can serve to support future investigations on the topic.
